# Use of stability statistics in the selection of *Clausena heptaphylla* (Roxb.) Wight & Arn for novel anethole rich strain (Jor Lab CH-2)

**DOI:** 10.3389/fpls.2022.1060492

**Published:** 2022-12-14

**Authors:** Mohan Lal, Sunita Munda, Anindita Gogoi, Twahira Begum, Joyashree Baruah, Sanjoy K. Chanda, Himangshu Lekhak

**Affiliations:** Agrotechnology and Rural Development (ARD) Division, CSIR-North East Institute of Science and Technology (NEIST), Jorhat, Assam, India

**Keywords:** selection breeding, GC/MS, multilocation trial, superior strain, stability

## Abstract

**Introduction:**

Anethole is an industrially important compound which is extensively used in pharmaceuticals, cosmetics, perfumery, food and confectioneries. Anethole is primarily obtained from fennel, anise, and star anise which is highly expensive. Therefore, a study was performed to identify a cost-effective and natural anethole rich strain of *Clausena heptaphylla* through selection and confirmed through multilocation trial.

**Methods:**

The study was conducted using 23 accessions collected from North eastern region of India from 2014-2018 (initial evaluation trial) and 2018-2022 (multilocation trial). The initial trial was conducted in the experimental farm of CSIR-NEIST, Jorhat, Assam using Complete Randomized Block Design with three replications. Five agronomical traits (plant height, leaf length, leaf width, number of stem branching, herbage yield per plant per cutting) along with essential oil yield and anethole content were evaluated which led to the identification of anethole rich strain of *C. heptaphylla*. This identified strain was further evaluated along with the two check genotypes for stability based on three yield parameters *viz.* herbage yield, essential oil yield and anethole content at four multi-locations (Imphal, Jorhat, Runne and Madang) for four years using the same experimental design.

**Results and discussion:**

The identified superior line (Jor Lab CH-2) showed consistent performance for the studied yield parameters across all the environments maintaining its superiority. The identified strain exhibited average herbage yield of 1.2 Kg/plant/cutting and essential oil yield of 1.22%. The GC-MS analysis of the essential oil depicted trans anethole as the major constituent (93.25%) followed by estragole (4.85%) while benzene, 1,2-dimethoxy-4-(1-propenyl Isoeugenol methyl ether and *cis*-anethole were the trace components. This is the first novel report of anethole rich variant of *C. heptaphylla* which has undergone multilocational trial over the years. Jor Lab CH-2 strain will open a new scope for the industries to isolate anethole from a different source in a cost-effective approach.

## Introduction

Herbal drugs, natural health products and secondary metabolites of medicinal plants are increasing enormously as people are realizing globally the valuable sources of medicinal plants and thereby focusing extensively more on conservation and sustainable use of medicinal plants. There is enumerable availability of medicinally important plant species worldwide where several species of the genus *Clausena* are used as traditional medicine for human ailment (Pandey et al., 2012). The genus *Clausena* includes more than 30 species namely *C.anisata*, *C. excavata*, *C. harmandiana*, *C. indica*, *C. heptaphylla* which are allocated all over tropical and subtropical areas (Guo et al., 2018). Among all, *Clausena heptaphylla* belonging to Rutaceae family is one of the important medicinal and aromatic shrubs. It is commonly known as panbahar which is distributed widely in South and South east Asia (Majumder and Rahman, 2016). *Clausena* species are basically self-pollinated plant and is bisexual in nature having chromosome number 2n = 4x = 36 (Mehra and Khosla, 1973). Morphologically, *C. heptaphylla* is a shrub or tree which measure up-to 2-4 m long, leaves are compound, alternate and spiral which emits most agreeable fragrance with petiole slightly marginate, terminal paniculate cymes in inflorescence with flower about 5 mm long, greenish yellow and glabrous. A detailed investigation revealed that *C. heptaphylla* posseses carbazole alkaloids like heptazolicine extracted from roots (Bhattacharyya et al., 1984), clausenalene extracted from bark stem (Bhattacharyya et al., 1993), clausnapin (Bhattacharyya et al., 1984), clausenal (Chakraborty et al., 1995), coumarins like lunamarin A and lunamarin B (Sohrab et al., 1999), clausmarin A (Sohrab et al., 2001), lunamarin C (Sohrab et al., 2002) extracted from leaves.

Ethnobotanical survey revealed that *Clausena heptaphylla* are beneficial for the cure of paralysis, ulcerated nose, headache and muscular pain. They are also putative to be used as diurectic, astringent, insecticide, tonic, vermifuge (Yusuf et al., 1994; Fakruddin et al., 2012) and have antiseptic properties (Begum et al., 2011). It is chewed with leaves of Piper beetle 2-3 times daily to cure digestive problem (Mannaf et al., 2013). The leaf part is also used in fever, remove nicotine addiction and foul odor from mouth (Hossan et al., 2009). The bark of the plant is used for curing cattle wounds and sprains (Sohrab et al., 2001). The plant essential oil and flower extracts are used for skin inflammation and ophthalmia (Agarwal, 1986; Lal et al., 2022). A recent report suggested that the aqueous extracts of *C. heptaphylla* leaves were used for relieving cigarette craving and anti-obesity (Jerajasin et al., 2014). Various experiments have been performed where it was found that this plant possess various antimicrobial, antifungal, antioxidant, antidiabetic properties (Sohrab et al., 2001; Fakruddin et al., 2012; Lal et al., 2022). 

In comparison with costly synthetic drugs which have adverse effects, the inclination towards extraction of essential oil for preparation of green medicine which is safe and dependable is increasing tremendously (Pandey et al., 2021). Many scientific reports suggested that essential oil has therapeutic proficiency in curing various diseases (Lal et al., 2021; Dutta et al., 2021). The previous report on composition of essential oil mentioned that *Clausena heptaphylla* possesses a predominant component known as anethole (Lockwood, 1984; Nath et al., 1996; Lal et al., 2022). Anethole act as a flavoring agent and is widely used in food industry, cosmetics, perfumery and pharmaceuticals industries (Lal et al., 2022). It has multiple propitious effects in human health such as anti-inflammatory, antidiabetic, immunomodulatory, anticarcinogenic, antithrombotic consequence (Aprotosoaie et al., 2016; Lal et al., 2022). Till now very few studies are found where a detailed survey has been done on anethole rich essential oil composition of *Clausena heptaphylla*. Recent report on the biological activity of *C. heptaphylla* essential oil revealed skin whitening effect, anti-diabetic, anti-inflammatory, antimicrobial, antihelminthic, antinociceptive, gastroprotective, sedative properties representatives which is non-toxic in nature (Marinov and Kuzmanova, 2015; Munda et al., 2019; Lal et al., 2022). Therefore, to meet the demand of the industries and owing to the wide application of biological friendliness of this species, a study was conducted to identify high anethole rich strain of *Clausena heptaphylla* which will be stable across different locations over the years. The ability of a genotype to maintain the consistency of the character at high or low yield in various environments or during various years is known as stability (Munda et al., 2020). Genotype stability performance is a prerequisite for identification of superior variety and must be carried out in many conditions since genotypes are greatly influenced by the environment, locations, and years (Gupta et al., 2015). Several stability models are developed to study the consistency of the genotypes out of which AMMI, GGE biplot and MTSI models are used in the study for proper validation of the results. Earlier no studies were performed to validate the superiority and consistency of anethole rich strain of *C. heptaphylla*, therefore, this can be considered as the first report for varietal development of anethole rich Jor Lab CH-2. The development of a plant variety with good and consistent performance under many environmental conditions is quite difficult for plant breeder. Therefore, the identification of this beneficial line can be contemplated as a novel and resourceful report as it will help in conservation of this species for future prospect help in betterment of farmers as well as pharmaceuticals industries thereby making the strain a good candidate for further drug development program. Further, Jor Lab CH-2 will open a new scope for the industries to isolate anethole from a different source in a cost-effective approach.

## Materials and methods

### Initial evaluation trial

The matured seeds of *Clausena heptaphylla* were collected in a total of 23 accessions from various parts of North East India in the year 2014 and planted in augmented design for an initial field evaluation trial in March 2014. The list and source of the collected germplasm are provided in the **Table S1**. The field trial was conducted in the experimental farm of CSIR-NEIST, Jorhat, Assam, India having GPS location of 26° 44’ 15.6948” N latitude, 94° 9’ 25.4628” E longitude and elevation 94 m above MSL. The texture of the soil was sandy loam with pH of 5.2. The climatic conditions of the experimental site were recorded with minimum temperature of 8.1°C, maximum temperature of 37.8°C; minimum relative humidity (%) of 64.3, maximum relative humidity (%) of 100; average annual rainfall of 2480 mm respectively during the evaluation period. The field trial’s plot was 3 x 2 m in size, with a 45 x 45 cm gap between each plant and line, respectively. The leaf specimens of the genotypes were used to prepare the herbarium voucher which was deposited at departmental herbarium of the institute. The data of an initial field evaluation trial for morphological, essential oil yield and its constituents were tracked and recorded in the year 2016, 2017 and 2018. The maturity of the species can be attained after two years; therefore, the leaves were harvested for three years after every four months. A high essential oil and high herbage yielding accession (RRLCH-2) was found after the review of the selection trial’s data which was later named as Jor Lab CH-2.

### Multilocation trials

The seeds of the selected line along with two check genotypes was further planted in four separate locations of Northeast India namely, Imphal (Manipur), Jorhat (Assam), Runne (Arunachal Pradesh) and Madang (Assam) for four years during 2018-2022. The agro-climatic conditions of the selected multilocations for the study are represented in **Table S2**. The trial was conducted in Randomized Complete Block Design (RCBD) with three replications. All the morphological as well as essential oil and its components were documented for two years (2021 and 2022) after maturity which were further analysed statistically to check its productivity, essential oil content and quality. A total of eight environments (coded: E1, E2, E3, E4, E5, E6, E7, and E8) were studied comprising of four locations for two-year evaluation.

### Data observation and collection

A total of five agronomical traits like plant height (cm), leaf length (cm), leaf width (cm), number of stem branching, herbage yield per plant per cutting (Kg) were tracked for the evaluation. The chemical profiling of the essential oil extracted from all the accessions were documented as per the standard protocol (Lal et al., 2022). For data collection, ten healthy and mature plants were randomly chosen from each genotype of each replication, eliminating the border plants. The average values were considered to be the final values.

### Isolation of essential oil and chemical profiling

The Clevenger apparatus (3000 mL) was used to isolate the essential oil from the leaves of *Clausena heptaphylla* (300 gm) for four hours at a temperature of 99⁰C. The standardized procedure was formulated by Clevenger (1928) which was modified by Lal et al. (2022). The essential oil was subjected to anhydrous sodium sulphate for absorption of moisture content present followed by preservation in the glass vial. The essential oil percentage (v/w) of the isolated essential oils was estimated using the following formula given by Munda et al. (2020):Essential oil (FWB) = $\frac{volume\;of\;essential\;oil\;isolated\;\left(mL\right)}{weight\;of\;fresh\;leaves\;used\;\left(g\right)}\times 100$

### Qualitative analysis of essential oil

The qualitative analysis was performed in the Sophisticated Analytical Instrument Facility of the institute (CSIR-NEIST, Jorhat) during all the studied years for the initial (2016, 2017 and 2018) as well as multilocation (2021 and 2022) evaluation. Thermo Scientific TRACE 1110 gas chromatograph instrument was used to assess the chemical profiling of *C.heptaphylla* leaf essential oil integrated with TG-WAXMS column and FID (Flame Ionization Detector). The column dimension was 60 m × 0.25 µm. The initial temperature of the oven was at 40°C for 2 minutes which was progressively escalated to 250°C at a rate of 5°C/min. The concluding temperature was maintained at 30°C/min for 30 min at 300°C. The carrier gas used was Helium gas at 1mL/min flow rate. The prepared sample was diluted at a ratio of 1:100 (v/v) in acetone with a split ratio of 1:20 for 1min. Similarly, Agilent technologies gas chromatograph mass spectroscopy instrument fused with HP-5MS silica capillary column and mass selective detector (MSD5975 C) was utilized for GC/MS. The dimension of HP-5MS column was 30 m × 0.25 mm i.d with film thickness of 0.25 µm. The range for the GC/MS scan was set at 45-650 amu. The conditions used for GC/MS were in accordance with the GC parameters used for GC analysis. The mass spectral data from NIST/Willey library was deployed to compare the detected peaks of the sample followed by confirmation with Kovat’s index on HP-5MS. The standards (Fluka and Sigma Aldrich, Germany) were run for authentication of the isolated compounds. The conditions for the GC used in the study were as per standardized by Lal et al. (2022).

### Statistical analysis

Analysis of variance (ANOVA) for both pooled and individual data were estimated for the multi-location trial for two years. The F-test was used for the significant analysis of ANOVA. The stability models like AMMI (Additive multiplicative mean interaction model), GGE (genotype+ genotype × environment) biplot and (MTSI) Multi trait stability index was measured through stability analysis by Olivoto et al. (2019) developed R-package “metan”. The consistent and better performing genotypes were identified by using these multivariate analyses.

The G x E interaction of AMMI model is given by:

*Y*_*ij*_ =µ+*g*_*i*_+*e*_*j*_ +

$$d_{ij}$$

where *Y*_*ij*_ is the yield of the genotype; μ is the grand mean; *g*_*i*_ is the deviation of the mean of genotype i from μ; *e*_*j*_ is the deviation of the environment j from μ

The GGE biplot was studied using the following equation:


$$\hat{Y_{ij}}-\mu =\;G_{i}+\;E_{j}+\;GE_{ij}$$

where $\hat{Y_{ij}}$ =average phenotype for genotype i in environment j; *μ* = overall constant; *G*_*i*_ = random influence of genotype i; *E*_*j*_ = fixed effect of environment j; and *GE*_*ij*_ =random effect of the genotype i versus environment j interaction

Similarly, the formula for MTSI is as follows:


$$\text{MTSI}={\left[\sum_{j=1}^{\text{f}}{\left({\text{F}}_{ij}-{\text{ F}}_{j}\right)}^{2}\right]}^{0.5}$$

Where F*_j_* = ideotype’s *j* ^th^ score; F*_ij_* = *i* ^th^ genotype’s *j* ^th^ score

## Results and discussion

All the five morphological data for *Clausena heptaphylla*, its essential oil and anethole content were recorded and evaluated after two years of plantation in the year 2016, 2017 and 2018. The range of different morphological and chemical characteristics of the collected germplasm of *C. heptaphylla* were depicted in **Table 1**. The outcome of the selection trial was the identification of superior genotype rich in anethole content which was later named as Jor Lab CH-2. The average quantitative and qualitative data of the anethole rich genotype Jor Lab CH-2 of *C. heptaphylla* are indicated in **Table 2**. The herbage yield per plant per cutting was found to be 1.2 Kg while the essential oil yield and anethole content were recorded to be 1.22% and 92.59% respectively. The leaf essential oil yield of *C. heptaphylla* of North eastern region was found to be 0.81% with methyl chavicol as the major (57.5%) component followed by anethole content of 40.3% (Nath and Bordoloi, 1992). Later, Nath et al., in 1996 reported leaf essential oil yield of 1.40% in flowering stage and 1.20% in fruiting stage. Similarly, Munda et al. (2019) also reported essential oil yield of 1.28% from the *C. heptaphylla* leaves which was in accordance to the present study. The essential oil isolated from the leaves of *C. heptaphylla* in the current study was observed to be similar with the earlier study.

**Table 1:** Range for morphological and chemical data of *C.heptaphylla* germplasm in initial evaluation and multilocation trial.

S. No.	Characters	Range	
		Initial evaluation trial	Multilocation trial
1.	Plant height (cm)	108-260	120 - 234
2.	Leaf length (cm)	4.6-15	4.3 - 8.6
3.	Leaf width (cm)	2.7-5.6	1.8 - 2.85
4.	No. of stem branching	1-7	2 - 6
5.	Herbage yield/plant/cutting (kg)	0.6-1.2	0.41 - 1.12
6.	Essential oil (%)	0.8-1.22	0.43 - 1.25
7.	Anethole (%)	52-92.59	40.84 – 93.25

**Table 2:** Average quantitative and qualitative data of the anethole rich genotype Jor Lab CH-2 of *C. heptaphylla* during the initial trial.

S. No.	Characters	Quantitative data
1.	Plant height (cm)	234 ± 2.48
2.	Leaf length (cm)	9.3 ± 1.06
3.	Leaf width (cm)	3.2 ± 0.68
4.	No. of stem branching	7 ± 0.92
5.	Herbage yield/plant/cutting (Kg)	1.2 ± 0.23
6.	Essential oil (%)	1.22 ± 0.08
7.	Anethole (%)	92.59 ± 0.12

The stability of the selected line was further analysed through multilocation trial conducted at four different places of North East India planted for four years to confirm its consistency across different environments. The two check genotypes i.e., CV-1 and CV-2 which are the local genotypes of Assam and Manipur respectively were considered for comparison due to the absence of registered *C. heptaphylla* varieties till date. The most essential requirement for validation of genotype consistency and variety development is the trial conducted in multi-locations or multi-years (Lal et al., 2017; Munda et al., 2020). There are different parameters to check the stability of the genotypes out of which AMMI (Additive multiplicative mean interaction model), GGE (genotype+ genotype × environment) biplot and MTSI (Multi trait stability index) was used to confirm the stability of the selected superior lines and the check genotypes. The multilocation data of the selected strain Jor Lab CH-2 and check genotypes CV-1 and CV-2 for the year 2021 and 2022 are depicted in the **Tables S3, S4**.

**Figure 1:**
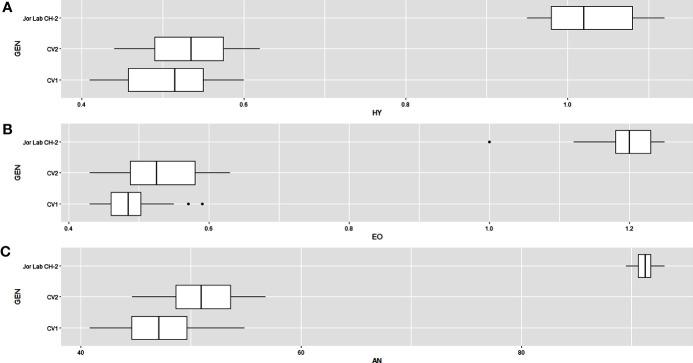
Box plots depicting the variation in **(A)** Herbage yield plant^-1^ (HY), **(B)** Essential oil yield (EO) and **(C)** Anethole content (AN) for individual genotypes over the environments. The dashed line shows the overall mean.

**Figure 2:**
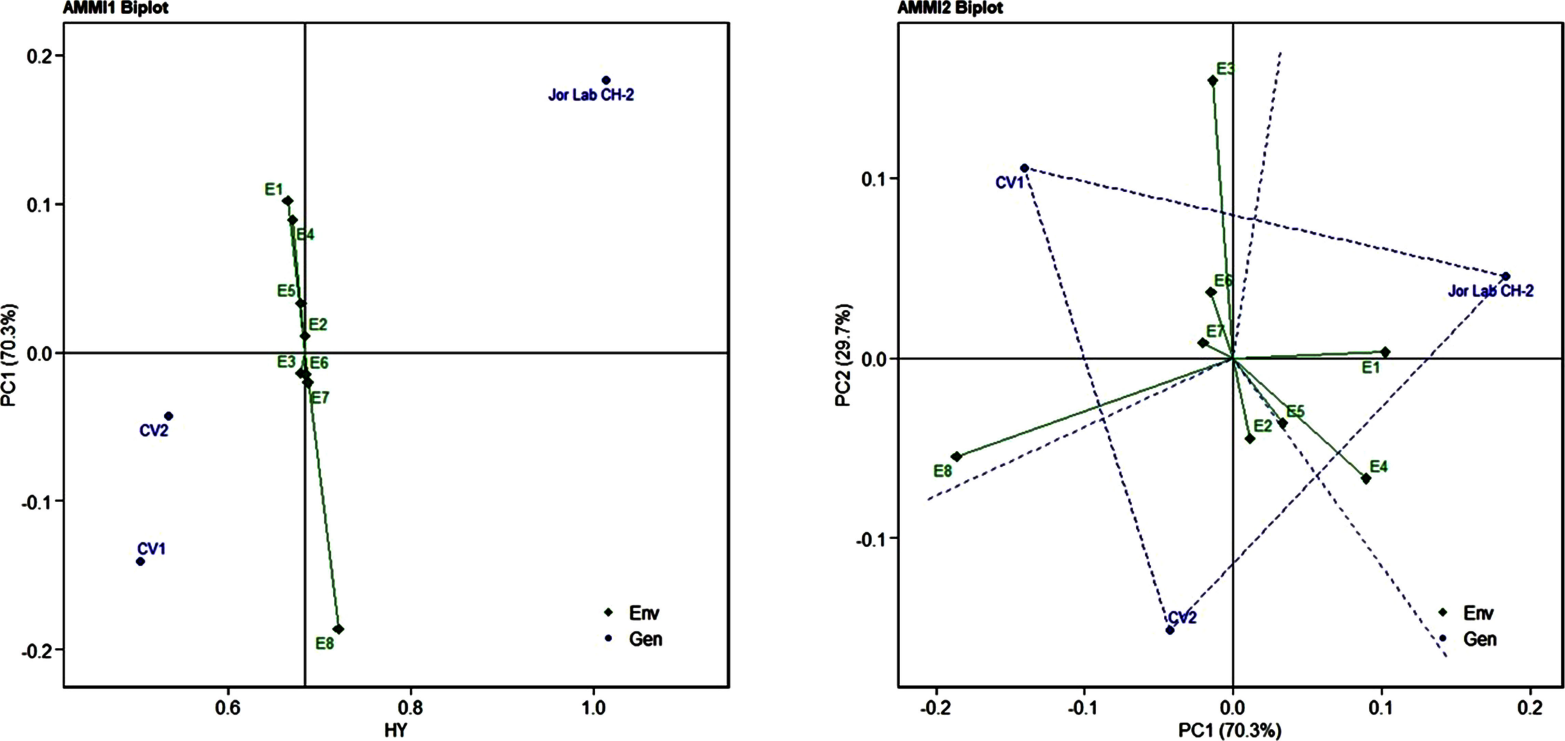
AMMI 1 and 2 model biplot for herbage yield/plant (Kg) of Jor Lab CH-2 compared with the check genotypes (CV1 and CV2) evaluated in eight environments.

**Figure 3:**
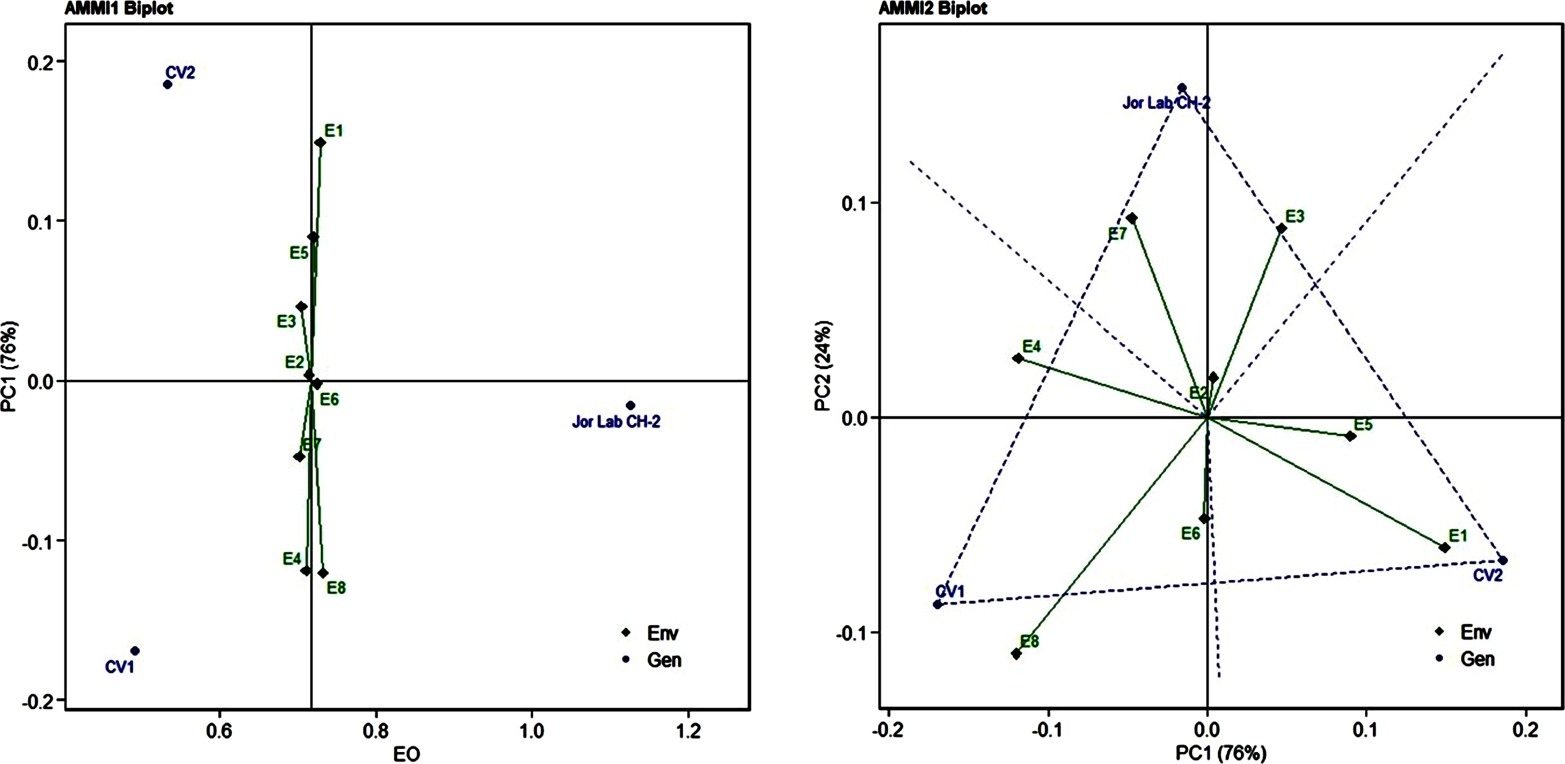
AMMI 1 and 2 model biplot for essential oil yield (%) of Jor Lab CH-2 compared with the check genotypes (CV1 and CV2) evaluated in eight environments.

**Figure 4:**
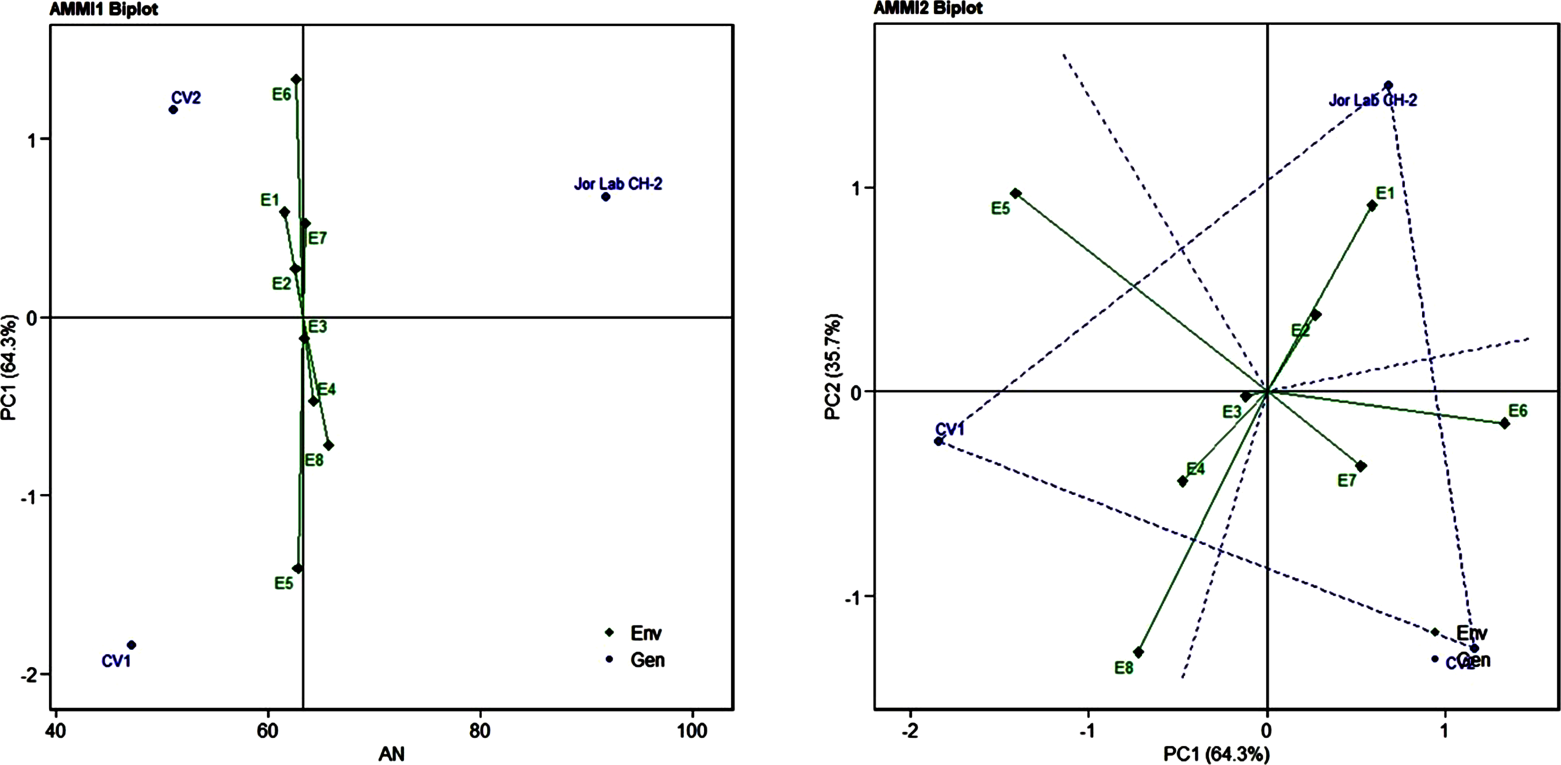
AMMI 1 and 2 model biplot for anethole content (%) of Jor Lab CH-2 compared with the check genotypes (CV1 and CV2) evaluated in eight environments.

**Figure 5:**
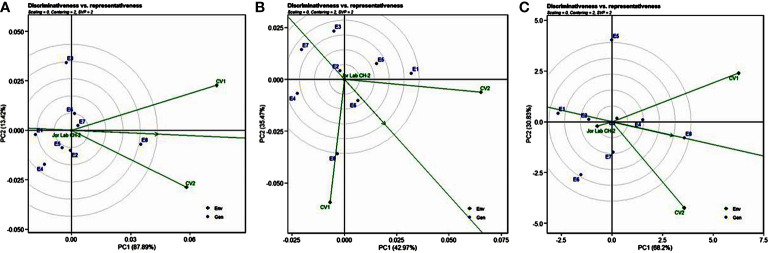
interaction effect of Jor Lab CH-2, CV1 and CV2 over two years across four multilocations for **(A)** Herbage yield plant^-1^ **(B)** Essential oil yield **(C)** Anethole content. The biplots were created based on Scaling = 0, Centering = 0,SVP = 2.

**Figure 6:**
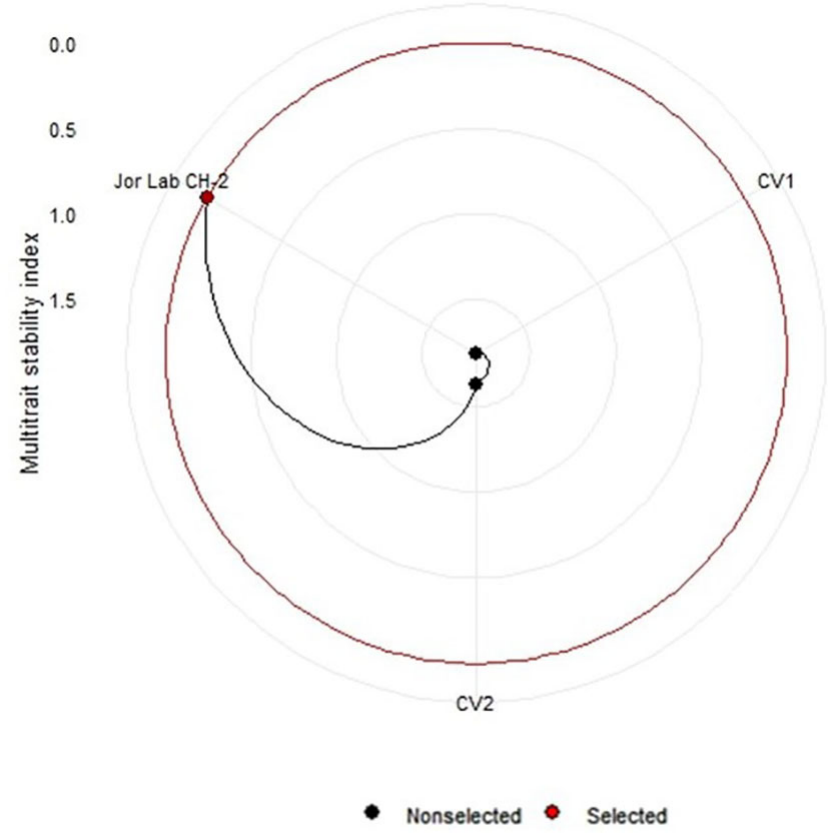
Multi trait stability index (MTSI) of Jor Lab CH-2 and check genotypes based on three yield parameters (Herbage yield plant^-1^, Essential oil yield and Anethole content).

Analysis of variance (ANOVA) was computed using the genotypes and multi-environment trial data which indicated that all the seven traits were statistically significant in the studied environments (E1, E2, E3, E4, E5, E6, E7, and E8). The genotypes were found to be statistically significant for all the studied traits. The genotype × environment (G × E) interaction was observed for the traits leaf width, herbage yield, essential oil and anethole content (**Table 3**). Since, G × E interaction was observed in the economic traits, therefore, stability analysis was performed for the three traits (HY, EO and AN). The variation observed in the three genotypes for the yield traits are represented as boxplots for herbage yield/plant/cutting (Kg), essential oil yield and anethole content (**Figure 1**). Boxplots are the representative of the concerned traits on the basis of variation, mean and range. The herbage yield (HY) varied from 0.41 (CV1 in E4) to 1.12 (Jor Lab CH-2 in E6) Kg per plant irrespective of different genotypes and multi-environments. Similarly, essential oil ranged from 0.43 (CV1 in E3) to 1.25% (Jor Lab CH-2 in E1) with CV of 44.25% and anethole content varied from 40.84 (CV1 in E7) to 93.25% (Jor Lab CH-2 in E1) with CV of 32.11%. The CV (coefficient of variation) was highest for essential oil yield (44.25%) followed by the number of stem branching per plant (41.03%) and herbage yield per plant (35.99%). The least variation was observed for leaf width (12.89%) with a range of 1.8–2.85 cm. The genotype variability in different environments was indicated for the studied traits. The AMMI ANOVA for herbage yield per plant depicted 97.85% of the total sum of squares (SS) was due to genotype, 0.78 and 0.47% due to environment and interaction between genotype-environment (GEI) respectively. Similarly, 98.64, 0.24, and 0.39% of the SS for essential oil yield was due to genotype, environment and GEI respectively. The anethole content revealed highest SS% due to genotype (98.88), lowest for environment (0.30) and GEI (0.49) (**Table 3**). The high value of SS% for genotype depicted strong influence of genotypes on the yield parameters while very low environmental influence can be predicted. High value for sum of squares is an indication of strong influence of the source as well as specifies its diverse nature (Alam et al., 2015; Singh et al., 2019). The interaction of genotype with the environment was partitioned into two principal components for all the studied traits. Therefore, the effects of environment as well as genotypes were illustrated simultaneously in biplots of AMMI1 (additive main effects vs IPCA1) and AMMI2 (IPCA1 vs IPCA2). The consistency and high yield of the selected superior line (Jor Lab CH-2) was clearly revealed in the AMMI biplots compared with the check genotypes (**Figures 2**–**4**). The key to successful breeding program for development of superior varieties is high yield together with the consistent performance of the genotype in different environments (Lal et al., 2020; Munda et al., 2021). The GGE biplot was also generated since it enables evaluation of environments due to discriminativeness and representativeness of genotype plus genotype-environment interaction which makes it superior to the AMMI biplot analysis (**Figure 5**) (Yan et al., 2007). Similarly, MTSI analysis clearly demonstrated that Jor Lab CH-2 is the most stable and high yielding genotypes based on all the yield and quality traits (**Figure 6**). MTSI is a unique technique for selecting stable preferred genotypes in crop breeding programmes based on multiple traits (Benakanahalli et al., 2021). All these parametric analysis supports the notion that the selected strain performed well in all the multi-locations over the years making it a novel anethole rich strain of *C. heptaphylla*. The suitable environment for the herbage yield, essential oil yield and anethole content were also determined in GGE biplot. The environment E7 followed by E6 showed best suitability for herbage yield while for essential oil yield (E2 followed by E6) and anethole content, environment E3 followed by E2 were the most favorable environments. The location Imphal and Madang located in Manipur and Assam respectively could be considered as the suitable location for Jor Lab CH-2.

**Table 3:** AMMI Analysis of variance for the seven traits in *C. heptaphylla* in MLT.

Source		Plant Height		Leaf Length		Leaf Width		Number of Stem Branching		Herbage Yield		Essential Oil %		Anethole content	
	DF	MS	SS%	MS	SS%	MS	SS%	MS	SS%	MS	SS%	MS	SS%	MS	SS%
ENV	7	87.6	613.2	0.109	0.760	0.032	0.221	0.159	1.111	0.005	0.033	0.003	0.019	12.57	88.0
REP (ENV)	16	31.4	502.7	0.329	5.274	0.048	0.773	0.917	14.667	0.002	0.038	0.003	0.055	5.83	93.3
GEN	2	64653.3	129306.7	67.334	134.668	1.578	3.156	43.931	87.861	2.072	4.145	3.709	7.419	14298.39	28596.
GEN × ENV	14	17.5	245.5	0.107	1.496	0.051	0.707	0.248	3.472	0.001	0.020	0.002	0.029	10.21	142.9
PC1	8	24.2	193.3	0.153	1.225	0.067	0.534	0.363	2.905	0.002	0.017	0.002	0.018	11.28	90.2
PC2	6	8.7	52.2	0.045	0.271	0.029	0.173	0.095	0.567	0.001	0.003	0.002	0.011	8.78	52.7
Residuals	32	36.6	1171.1	0.274	8.753	0.044	1.394	0.667	21.333	0.004	0.113	0.002	0.073	7.60	243.2
Total	85	1553.9	132084.6	1.794	152.447	0.082	6.959	1.522	131.917	0.051	4.369	0.089	7.623	344. 79	29307.1
CV		25.95		23.35		12.89		41.03		35.99		44.25		32.11	

ENV, environment; REP, replication; GEN, genotype; PC, Principal components; DF, degree of freedom; MS, mean sum of squares; CV, coefficient of variation.

**Figure 7:**
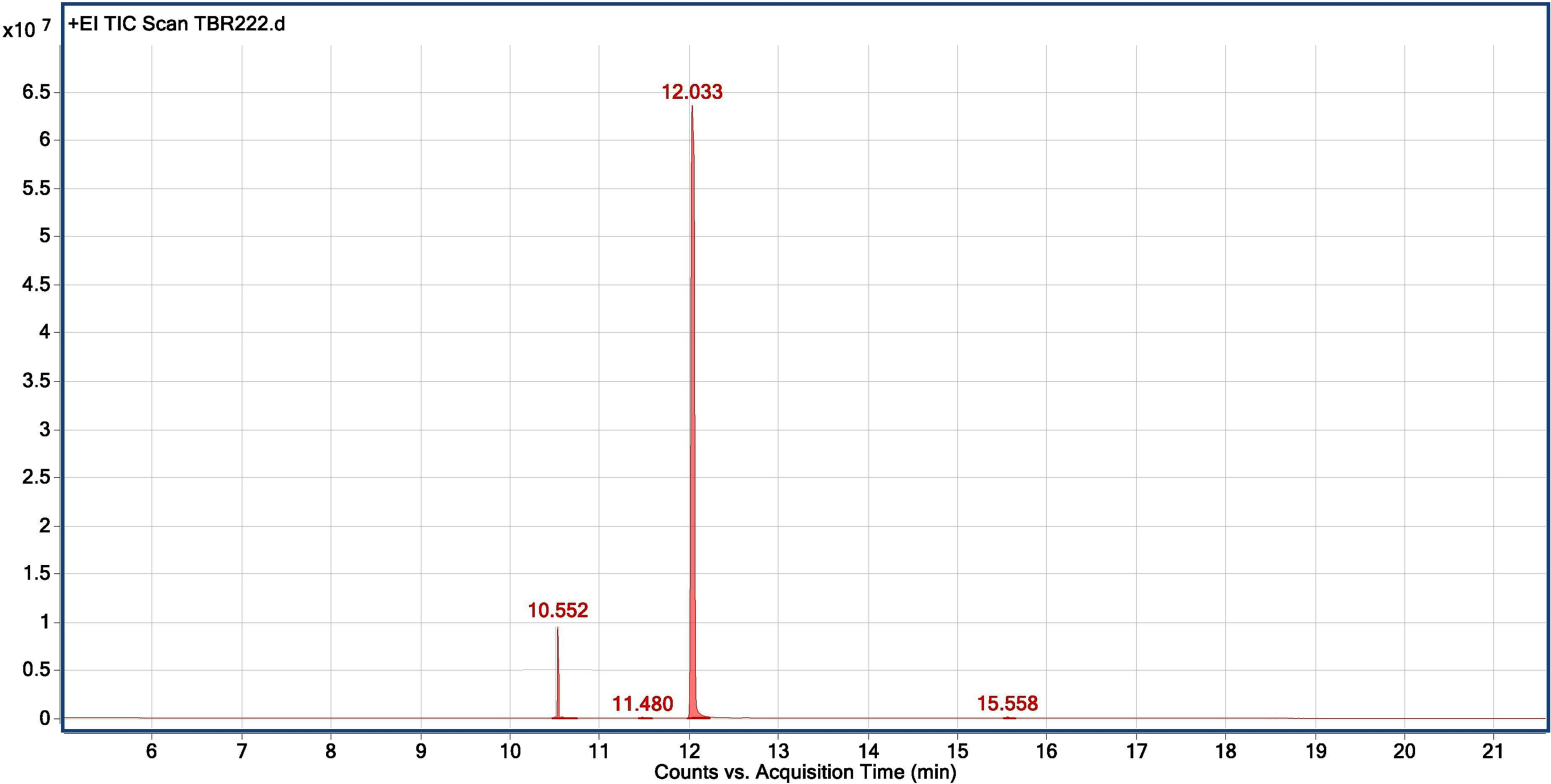
Chromatogram for GC-MS analysis of anethole rich variety Jor Lab CH-2 of *Clausena heptaphylla*.

The chemical profiling of the essential oil analysis for Jor Lab CH-2 led to the identification of four compounds accounting for 98.54% of the total essential oil (**Figure 7**). The compound trans-Anethole was the principal constituent with area percentage of 93.25% which was followed by estragole (4.85%). The trace components were cis-Anethole (0.27%) and Benzene, 1,2-dimethoxy-4-(1-propenyl Isoeugenol methyl ether (0.17%) (**Table 4**). Trans anethole is an ethanol soluble volatile compound characterized by grassy and sweet aroma. It is an isomer of E-anethole and is widely used in pharmaceutical, cosmetic, food and fragrance industries (Akcan et al., 2018). Anethole is dominantly present in the essential oil of *Illicium verum* (72-92%) and also in *Foeniculum vulgare* and *Pimpinella anisum* seed (Sharafan et al., 2022). In contrast, the present study demonstrated that the superior strain of *Clausena heptaphylla* essential oil exhibited an average of 92.59% trans anethole which is higher than the *Illicium verum* essential oil. The essential oil of *I. verum*, *F. vulgare* and *P. anisum* seeds are very expensive which could be substituted with the easily available *C. heptaphylla* proving to be a cost-effective alternative source. The essential oil of the identified strain Jor Lab CH-2 can be a good source of anethole for industrial applications due to its various biological activities as pharmaceutical applications. A recent study reported the efficiency of anethole rich Jor Lab CH-2 as skin whitening agent, anti-diabetic and anti-inflammatory representatives which is non-toxic in nature (Lal et al., 2022). Similarly, other pharmacological applications include antimicrobial, anti-helminthic, antinociceptive, gastroprotective, sedative properties (Marinov and Kuzmanova, 2015; Munda et al., 2019). Additionally, it also serves as a masking agent to cover up unpleasant odors in various products like mouthwash, toothpaste, soaps etc. It is also used in food industry as flavoring agent and additives in chewing gums, candies, baked foods etc. Therefore, the anethole rich strain of *C. heptaphylla* will be highly beneficial to the essential oil, food, perfumery and pharmaceutical industries. Earlier studies from North Eastern region of India performed on *C. heptaphylla* essential oil revealed only 40.3% E-anethole in leaf essential oil and 12.7% in fruit essential oil which is much lower to the present strain (Nath and Bordoloi, 1992). Later, Nath et al. (1996) reported the presence of E-anethole as the major component in *C. heptaphylla* essential oil with 98.2% in leaf essential oil at both fruiting and flowering stage while 61.67% anethole was revealed in fruit essential oil. Some investigation also reported that other species of *Clausena* like *C. anisate*, *C. austroindica* and *C. harmandiana* possesses anethole (80.00–97.44%) as major component in leaf essential oil (Addae-Mensah et al., 1996; Johnson et al., 2022). Another report mentioned that *C. indica* essential oil was rich in myristicin (35.3%) content (Diep et al., 2009) while *C. pentaphylla* yielded 0.80% essential oil having methyl eugenol (38.1%) as the principal volatile constituent (Pandey et al., 2012). The variation in the chemical constituents of the essential oil may serve as the chemotaxonomic value for intraspecific differentiation of *Clausena* species. A recent report was published by the same team on the biological activities of Jor Lab CH-2 which indicated the presence of anethole (88.59%) as the principal component (Lal et al., 2022). However, the stability analysis was not performed to observe its consistency across different locations over the years which is an essential criterion for varietal development. The strain clearly represented its stability based on herbage yield, essential oil yield and anethole content across four different multi-locations of NE India.

**Table 4:** GC-MS analysis of anethole rich variety of *C. heptaphylla* (Jor Lab CH-2) leaf essential oil planted at Madang location.

Compound	Retention Time	Retention Indices^exp^	Retention Indices^lit^	Area%
Estragole	10.55	1196	1198	4.85
cis-Anethole	11.48	1254	1256	0.27
trans-Anethole	12.03	1284	1285	93.25
Benzene, 1,2-dimethoxy-4-(1-propenyl Isoeugenolmethyl ether	15.55	1492	1492	0.17
Total identified				98.54
Total identified				1.46
Total				100.00

exp, experimental; lit, literature.

## Conclusions

*Clausena heptaphylla* is a plant of industrial importance because the essential oil of this plant consists of anethole which is used in pharmaceutical, perfumery and food industries. Currently, the industries rely on *Illicium verum*, *Foeniculum vulgare* and *Pimpinella anisum* seeds to produce anethole but the essential oil of these species are very expensive. Therefore, the essential oil of these species can be substituted with Jor Lab CH-2 which is anethole rich strain and cost effective. This high anethole rich strain of *C. heptaphylla* was identified through evaluation trial for four years followed by multilocation trial in four different locations for four years with a total of eight-year study. The agronomical and the biochemical data clearly indicated that Jor Lab CH-2 was superior than the other studied genotypes with plant height of 234 cm, leaf length (9.3 cm), leaf width (3.2 cm), number of stem branching (7), herbage yield of 1.2 Kg per plant, essential oil% (1.22%) and anethole% (92.59%). Stability along with the superiority is an important step in varietal development programme; therefore, stability parameters like AMMI, GGE and MTSI were used to confirm the results of the identified strain along with the check genotypes indicating the stable performance of Jor Lab CH-2 in terms of economic yield. This is the first report on the identification of anethole rich novel strain of *C. heptaphylla* which was followed by stability analysis, the results were found to be consistent and satisfactory. This new strain will expand the possibilities for the commercial production of anethole and offer the pharmaceutical, perfumery and food industries new cheap sources of raw materials.

## Data availability statement

The original contributions presented in the study are included in the article/**Supplementary Material**, further inquiries can be directed to the corresponding author.

## Author contributions

ML: Conceptualization, methodology, supervision, resources, review and editing. SM: Investigation, methodology, formal and software analysis, writing - original draft. AG: Writing, formatting and editing. TB: GC-MS analysis of essential oil. JB: Data curation. SC: Formal analysis. HL: Data curation. All authors contributed to the article and approved the submitted version.
