# Supplementary material for: Use of stability statistics in the selection of Clausena heptaphylla (Roxb.) Wight & Arn for novel anethole rich strain (Jor Lab CH-2)
Source: Front Plant Sci. 2022 Dec 14;13:1060492. doi: 10.3389/fpls.2022.1060492 (PMC9794615; doi:10.3389/fpls.2022.1060492)
Supplement: Supplementary file 1 [file DataSheet_1.docx]

Table S1. The code list and collection site of *Clausena heptaphylla* germplasm used in the study

| Code | Location/District/State |
| --- | --- |
| RRLCH-1 | Makum/Tinsukia/Assam |
| RRLCH-2 | Nagazanka/Jorhat/Assam |
| RRLCH-3 | Gargaon, Sivasagar/ Assam |
| RRLCH-4 | Sepon/ Sivasagar/ Assam |
| RRLCH-5 | Charaideo/ Charaideo/Assam |
| RRLCH-6 | Gemon/Sivasagar/ Assam |
| RRLCH-7 | Aalo/ West Siang/ Arunachal Pradesh |
| RRLCH-8 | Pasighat/ East Siang/ Arunachal Pradesh |
| RRLCH-9 | Pasighat/ East Siang/ Arunachal Pradesh |
| RRLCH-10 | Sonari/ Sivasagar/ Assam |
| RRLCH-11 | Itanagar/Itanagar, Arunachal Pradesh |
| RRLCH-12 | Ziro/ Lower Subansiri/ Arunachal Pradesh |
| RRLCH-13 | Sonari/ Sivasagar/ Assam |
| RRLCH-14 | Borpathar/ Golaghat/Assam |
| RRLCH-15 | Dhekial/ Golaghat/ Assam |
| RRLCH-16 | Gauripur/Dhubri/Assam |
| RRLCH-17 | Moran/ Dibrugarh/ Assam |
| RRLCH-18 | Longso/ Karbi Anglong/ Assam |
| RRLCH-19 | Shilonijan/ Karbi Anglong/ Assam |
| RRLCH-20 | Morigaon/ Morigaon/ Assam |
| RRLCH-21 | Boko/Kamrup/ Assam |
| RRLCH-22 | Dimapur/ Dimapur/ Nagaland |
| RRLCH-23 | Wokha/ Wokha/ Nagaland |

Table S2. Agro-climatic condition of the different multilocation sites (2018-2022)

| Locations | Min Temperature | Max Temperature | Min Relative Humidity | Max Relative Humidity | Average Rainfall |
| --- | --- | --- | --- | --- | --- |
| Imphal (Manipur) | 9°C | 34.2°C | 42 % | 100 % | 1680 mm |
| Jorhat (Assam) | 8.8°C | 41°C | 42 % | 100 % | 2244 mm |
| Runne (Arunachal Pradesh) | 7.2°C | 35.4° C | 40 % | 100 % | 2840 mm |
| Madang (Assam) | 8.8° C | 36 °C | 47.3% | 100 % | 2345 mm |

Table S3. The Multilocation trial data of Jor Lab CH-2 along with the check strains for the year 2021

| Variety | Location | Plant height (cm) | Leaf length (cm) | Leaf width (cm) | No. of stem branching | Herbage yield/plant/cutting (Kg) | Essential oil% | Anethole content% |
| --- | --- | --- | --- | --- | --- | --- | --- | --- |
| Jor Lab CH-2 | Imphal | 226 | 7.9 | 2.8 | 3 | 1.00 | 1.20 | 90.83 |
|  | Jorhat | 230 | 8.6 | 3.0 | 5 | 1.01 | 1.21 | 91.72 |
|  | Runne | 226 | 8.2 | 2.5 | 3 | 0.98 | 1.19 | 91.08 |
|  | Madang | 228 | 8.0 | 2.8 | 4 | 1.01 | 1.20 | 91.26 |
|  | Mean | 227.50 | 8.18 | 2.78 | 3.75 | 1.00 | 1.20 | 91.22 |
|  | SD | 1.66 | 0.27 | 0.18 | 0.83 | 0.01 | 0.01 | 0.29 |
| CV-1 (Check-1) | Imphal | 128 | 5.3 | 2.2 | 2 | 0.47 | 0.48 | 44.01 |
|  | Jorhat | 132 | 5.2 | 2 | 3 | 0.50 | 0.49 | 45.74 |
|  | Runne | 120 | 4.8 | 1.8 | 3 | 0.52 | 0.46 | 47.42 |
|  | Madang | 143 | 5.5 | 2.6 | 3 | 0.47 | 0.50 | 49.03 |
|  | Mean | 130.75 | 5.20 | 2.15 | 2.75 | 0.49 | 0.48 | 46.55 |
|  | SD | 8.29 | 0.25 | 0.29 | 0.43 | 0.02 | 0.01 | 1.87 |
| CV-2 (Check-2) | Imphal | 142 | 5 | 2 | 1 | 0.51 | 0.58 | 48.85 |
|  | Jorhat | 146 | 5.5 | 2.1 | 2 | 0.54 | 0.53 | 50.17 |
|  | Runne | 137 | 5.1 | 2.3 | 2 | 0.51 | 0.52 | 51.08 |
|  | Madang | 152 | 5.6 | 2.8 | 2 | 0.53 | 0.50 | 52.05 |
|  | Mean | 144.25 | 5.30 | 2.30 | 1.75 | 0.52 | 0.53 | 50.54 |
|  | SD | 5.49 | 0.25 | 0.31 | 0.43 | 0.01 | 0.03 | 1.18 |
| Grand Mean | | 167.5 | 6.23 | 2.41 | 2.75 | 0.67 | 0.74 | 62.77 |
| SD | | 43.18 | 1.40 | 0.38 | 1.01 | 0.23 | 0.33 | 20.23 |
| LSD | | 7.84 | 0.35 | 0.46 | 0.9 | 0.02 | 0.03 | 1.74 |
| F-value | | 243.15 | 127.29 | 4.47 | 8.47 | 1087.33 | 1283.8 | 1099.85 |
| CV% | | 0.26 | 0.22 | 0.16 | 0.37 | 0.34 | 0.46 | 0.32 |

Table S4. The Multilocation trial data of Jor Lab CH-2 along with the check strains for the year 2022

| Variety | Location | Plant height (cm) | Leaf length (cm) | Leaf width (cm) | No. of stem branching | Herbage yield/plant/cutting (Kg) | Essential oil% | Anethole content% |
| --- | --- | --- | --- | --- | --- | --- | --- | --- |
| Jor Lab CH-2 | Imphal | 223 | 8.07 | 2.41 | 3 | 1.00 | 1.20 | 90.72 |
|  | Jorhat | 224 | 8.66 | 2.82 | 4 | 1.02 | 1.21 | 91.23 |
|  | Runne | 226 | 6.84 | 2.49 | 4 | 1.00 | 1.19 | 91.08 |
|  | Madang | 223 | 8.3 | 2.85 | 4 | 1.01 | 1.20 | 90.65 |
|  | Mean | 224 | 7.97 | 2.64 | 3.75 | 1.01 | 1.20 | 90.92 |
|  | SD | 1.22 | 0.68 | 0.19 | 0.43 | 0.01 | 0.01 | 0.24 |
| CV-1 | Imphal | 132 | 5.38 | 2.32 | 4 | 0.49 | 0.48 | 48.95 |
|  | Jorhat | 135 | 5.22 | 2.03 | 3 | 0.51 | 0.50 | 43.99 |
|  | Runne | 128 | 4.87 | 1.85 | 2 | 0.51 | 0.48 | 46.39 |
|  | Madang | 147 | 5.61 | 2.59 | 3 | 0.56 | 0.54 | 51.12 |
|  | Mean | 135.50 | 5.27 | 2.20 | 3.00 | 0.52 | 0.50 | 47.61 |
|  | SD | 7.09 | 0.27 | 0.28 | 0.71 | 0.03 | 0.02 | 2.68 |
| CV-2 | Imphal | 147 | 5.06 | 2.05 | 2 | 0.53 | 0.55 | 47.71 |
|  | Jorhat | 150 | 5.52 | 2.21 | 3 | 0.53 | 0.54 | 52.15 |
|  | Runne | 145 | 5.26 | 2.37 | 2 | 0.54 | 0.50 | 52.33 |
|  | Madang | 158 | 5.67 | 2.79 | 3 | 0.59 | 0.53 | 54.23 |
|  | Mean | 150 | 5.38 | 2.36 | 2.50 | 0.55 | 0.53 | 51.61 |
|  | SD | 4.95 | 0.23 | 0.28 | 0.5 | 0.02 | 0.02 | 2.39 |
| Grand Mean | | 169.83 | 6.21 | 2.40 | 3.08 | 0.69 | 0.74 | 63.38 |
| SD | | 39.08 | 1.32 | 0.31 | 0.76 | 0.23 | 0.32 | 19.65 |
| LSD | | 6.79 | 0.61 | 0.61 | 0.97 | 0.03 | 0.02 | 2.8 |
| F-value | | 265.94 | 35.25 | 2.37 | 3.80 | 502.83 | 1566.5 | 397.92 |
| CV% | | 0.23 | 0.21 | 0.13 | 0.25 | 0.33 | 0.43 | 0.31 |
